# Identification of significant modules and hub genes involved in hepatic encephalopathy using WGCNA

**DOI:** 10.1186/s40001-022-00898-3

**Published:** 2022-11-24

**Authors:** Chihao Zhang, Guqing Luo, Jiayun Lin, Zhifeng Zhao, Meng Luo, Hongjie Li

**Affiliations:** Department of General Surgery, Shanghai Ninth People’s Hospital, School of Medicine, Shanghai Jiao Tong University, 639 Zhi Zao Ju Road, Huangpu District, Shanghai, 200011 China

**Keywords:** WGCNA, Hepatic encephalopathy, Neuroinflammation, CYBB, FOXO1

## Abstract

**Background:**

Hepatic encephalopathy (HE) is a reversible syndrome of brain dysfunction caused by advanced liver disease. Weighted gene co-expression network analysis (WGCNA) could establish a robust co-expression network to identify the hub genes and underlying biological functions. This study was aimed to explore the potential therapeutic targets in HE by WGCNA.

**Results:**

The green and brown modules were found to be significantly associated with the development of HE. Functional enrichment analyses suggested the neuroinflammation, neuroimmune, extracellular matrix (ECM), and coagulation cascade were involved in HE. CYBB and FOXO1 were calculated as hub genes, which were upregulated in the HE patients. Tamibarotene and vitamin E were suggested as possible drug candidates to alleviate HE.

**Conclusions:**

It is the first time to analyze transcriptomic data of HE by WGCNA. Our study not only promoted the current understanding of neuroinflammation in HE, but also provided the first evidence that CYBB and FOXO1 played pivotal roles in the pathogenesis of HE, which might be potential biomarkers and therapeutic targets. Tamibarotene might be a novel drug compound against HE.

**Supplementary Information:**

The online version contains supplementary material available at 10.1186/s40001-022-00898-3.

## Background

Hepatic encephalopathy (HE) is a common but serious complication of advanced liver disease due to liver insufficiency, which is accompanied by a spectrum of neuropsychiatric manifestations and leads to high morbidity and mortality [1–3]. Therefore, effective therapeutic strategies are essential to improve the prognosis of HE.

In-depth understanding of pathophysiology in HE is the basis for effective therapeutic strategies. Hyperammonemia remains the central pathophysiological process in the pathogenesis of HE. However, previous studies have highlighted the limited predictive value of blood ammonia in HE, which suggested the existence and importance of other factors [4, 5]. Besides hyperammonemia, overwhelming evidence has demonstrated the important role of systemic and central inflammation, malnutrition, gut–liver–brain axis, and neurotransmitters during the development of HE [1, 5, 6].

During the past decades, an emerging role of neuroinflammation in the development of HE has been demonstrated [7, 8]. As a key pathogenic factor in HE, neuroinflammation can be induced by inflammatory cytokines, chemokines, and oxidative stress, which involves various cell types in brain including glial cells and peripherally recruited immune cells [1, 8, 9]. Meanwhile, several anti-inflammatory strategies were proven to be effective in ameliorating HE. Rifaximin protected against HE in cirrhotic patients by modulating gut-derived inflammation [10]. Babao Dan exerted anti-inflammatory effects and improved clinical minimal HE [11]. However, those available therapies were proved to be mainly against peripheral inflammation. Novel strategies were still lacking to directly alleviate the central inflammation. Moreover, most of anti-inflammatory therapies nonspecifically suppressed the inflammatory response, while targeted therapies were lacking to precisely impede the development of HE. Therefore, seeking for novel therapeutic targets against central inflammation is an important issue.

In recent years, a powerful method for systematic analysis called weighted gene co-expression network analysis (WGCNA) has been widely applied in bioinformatic analysis of various diseases [12]. The robust co-expression network could cluster genes with similar expression patterns into modules to identify the underlying biological functions. Moreover, the hub gene identification would reduce the bias brought by confounding factors, offering insight into potential therapeutic targets of the interested diseases. Therefore, in the current study, we used WGCNA to identify the clinically significant modules and hub genes of HE in cirrhotic patients, which might provide further evidence for novel therapeutic targets of HE.

## Results

### Construction of the HE co-expression network and identification of the modules

**Fig. 1:**
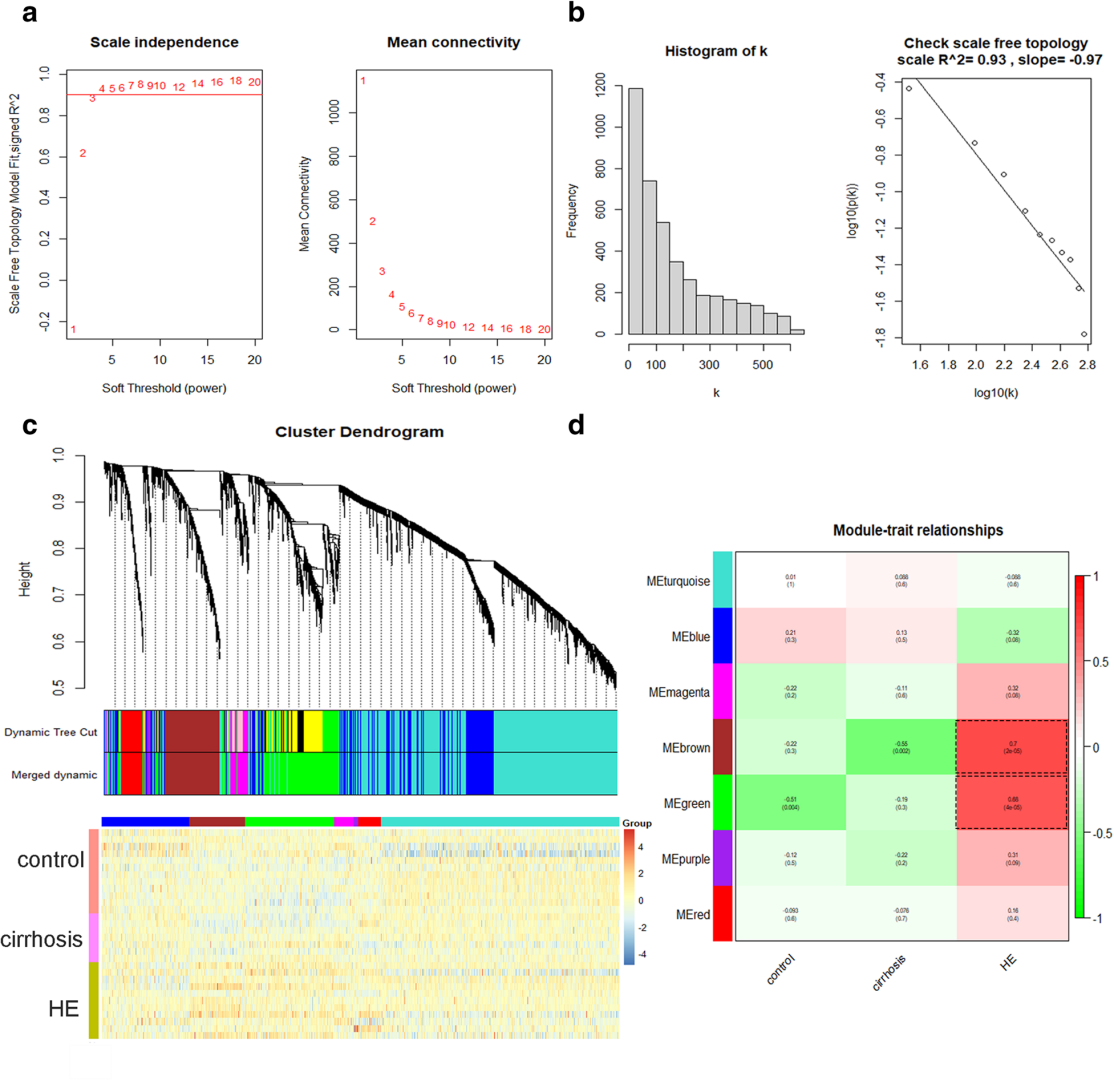
Construction of WGCNA. **a** Analyses of network topology for various soft-thresholding powers (weighting coefficient β), and the scale-free topology were set as 0.9 roughly. **b** Histogram of connectivity distribution, and the scale-free topology (*R*^2^ = 0.93) when *β* = 4. **c** Clustering dendrogram of the genes involved. **d** Heatmap of the correlations between MEs and clinical traits. Correlation coefficients and corresponding *p* values are shown in the rectangles and the brackets, respectively

After the removal of the outlier sample (GSM1027458) by clustering analysis, a total of 30 samples were involved in the WGCNA, including 12 healthy control samples, 7 cirrhosis samples, and 12 HE samples (Additional file 1: Figure S2). A total of 16,416 genes were annotated, when 25% of the genes with the greatest variance were selected to construct the co-expression network. Based on the 4104 selected genes, the optimal soft-thresholding power β was selected as 4 to ensure the scale-free topology (Fig. 1A). When the power was 4, the scale-free topology (R^2^) was 0.93 (Fig. 1B). After the optimal soft-thresholding power β was selected, the adjacency matrix of selected genes was constructed and subsequently transformed to TOM. Thus, dissTOM of 4104 selected genes was obtained and shown by the network heatmap plot (Additional file 1: Figure S1). Then, the modules with a minimum size of 30 were identified by the dynamic tree cut method (Fig. 1C). All 4104 genes were divided into certain clusters, while no gene was assigned to the grey module. Moreover, the average linkage hierarchical clustering was performed based on the average distance between the modules. Finally, seven modules of co-expressed genes were obtained after merging similar modules with a merging threshold function at 0.25 (Fig. 1C).

### Identification of the HE modules

**Fig. 2:**
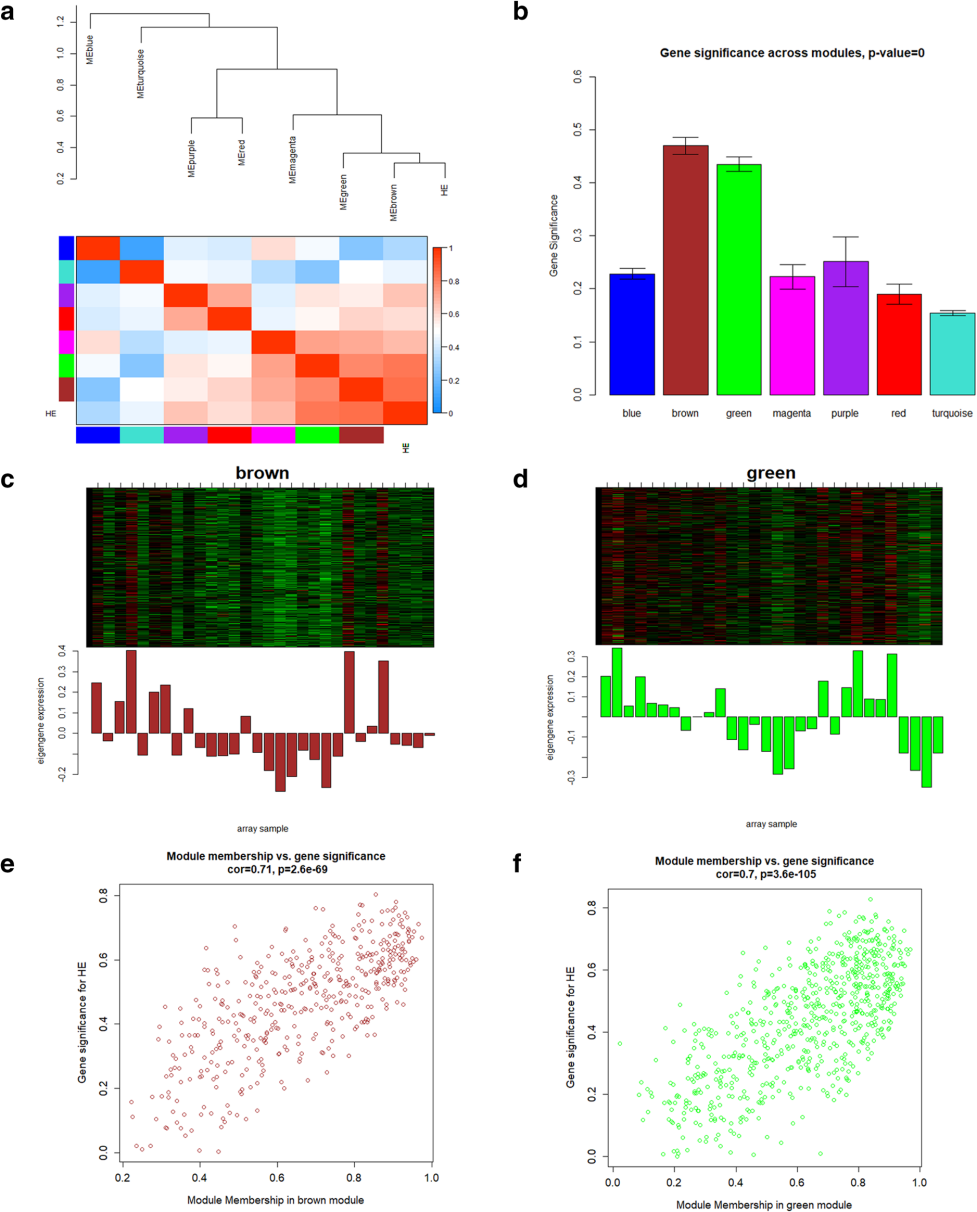
Identification of clinically significant modules. **a** Hierarchical clustering dendrogram of MEs and HE. Heatmap plot shows the adjacencies among them. Red color indicates positive correlation, while blue color indicates negative correlation. **b** Bar plots of mean absolute values of GS across modules. Higher mean GS suggests more significant associations between the module and HE. **c**, **d** The upper plots show the expression levels of all genes in the brown or green module (*y*-axis) among all samples (*x*-axis). The lower plots show the corresponding MEs (*y*-axis) versus the samples (x-axis). **e**, **f** Scatterplots of GS for HE versus MM in the brown or green module

After merging similar modules, the MEs of the seven obtained modules were calculated (Fig. 2C, D, Additional file 1: Figure S3A-E). To identify the clinically significant modules associated with HE, the Pearson’s correlations between the MEs of the seven modules and the clinical traits were obtained (Fig. 1D). According the module–trait relationships, the brown module (*r* = 0.7, *P* = 2e−05) and the green module (*r* = 0.68, *P* = 4e−05) were strongly associated with HE (Fig. 1D). Meanwhile, according to the correlations between the MEs and the interested clinical trait HE, a hierarchical clustering dendrogram and a heatmap were constructed, indicating that the brown module and the green module were tightly correlated to HE (Fig. 2A). Moreover, GS for HE was also calculated in each module. The mean absolute values of GS in each module were calculated and visualized, suggesting that the brown and green modules were the clinically significant modules with the highest GS for HE (Fig. 2B). Furthermore, the MM values were obtained, followed by the correlation analysis between the GS for HE and the MM for genes in the seven modules, respectively (Fig. 2E, F, Additional file 1: Figure S4A-E). With a comprehensive consideration of the correlation coefficients and the p-values, the brown module (*r* = 0.71, *P* = 2.6e−69) and the green module (*r* = 0.7, *P* = 3.6 e−105) were selected as clinically significant modules, which were consistent with the above results (Fig. 2E, F). Taken together, with three different approaches, we could conclude that the brown module and the green module were HE modules exhibiting the strongest associations with HE. Otherwise, the functional enrichment analysis of the other five modules did not achieve satisfactory results. Thus, we would focus on these two HE modules in the following investigations to further identify the biological functions and hub genes.

### Functional enrichment analysis and hub genes identification of the brown module

**Fig. 3:**
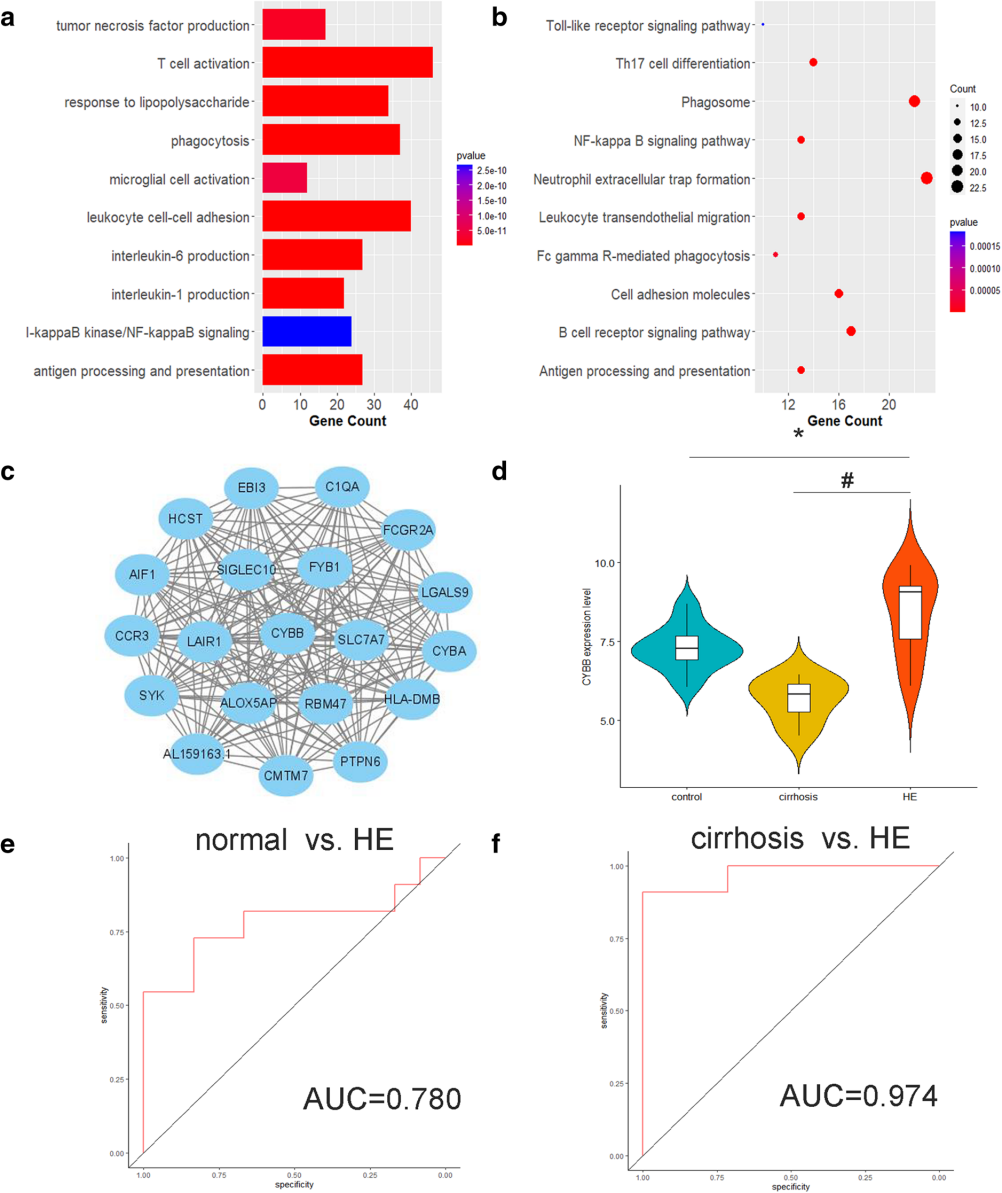
Functional enrichment analysis and hub genes identification of the brown module. **a** Bar chart of the top 10 significant terms of BP in GO enrichment analysis of the brown module. **b** Bubble diagram of the top 10 enriched pathways in KEGG pathway enrichment analysis of the brown module. **c** Network depiction of the brown module. The top 20 genes with the highest IC were involved. **d** Violin plots with included boxplots of CYBB expression levels among three groups. **e** ROC analysis to evaluate expression level of CYBB in predicting cirrhosis. f ROC analysis to evaluate expression level of FOXO1 in distinguishing HE from cirrhosis

Based on the clustered genes in the brown module, functional enrichment annotations were identified by GO enrichment analysis and KEGG pathway enrichment analysis. The top 10 significant terms of BP in GO enrichment analysis were visualized, suggesting that the genes in the brown module were associated with neuroinflammation- and neuroimmune-related biological processes (Fig. 3A). For KEGG pathway enrichment analysis, the top 10 enriched pathways were mainly related to neuroinflammation and neuroimmune as well (Fig. 3B). To identify the hub gene in the brown module, top 20 genes with the highest IC were extracted to construct the key network, which were defined as central genes. The connections among the central genes were visualized (Fig. 3C, Additional file 1: Table S1). Finally, CYBB was selected as the hub gene of the brown module as it had the highest degree of connections among 20 genes. Further analysis focusing on CYBB was performed. It was demonstrated that the expression level of CYBB was significantly upregulated in HE samples when compared to health control samples and cirrhosis samples, respectively (Fig. 3D). In addition, ROC analysis highlighted that CYBB could predict the diagnosis of HE (AUC = 0.78) (Fig. 3E). Meanwhile, CYBB was also capable of distinguishing between HE and cirrhosis (AUC = 0.974) (Fig. 3F).

### Functional enrichment analysis and hub genes identification of the green module

**Fig. 4:**
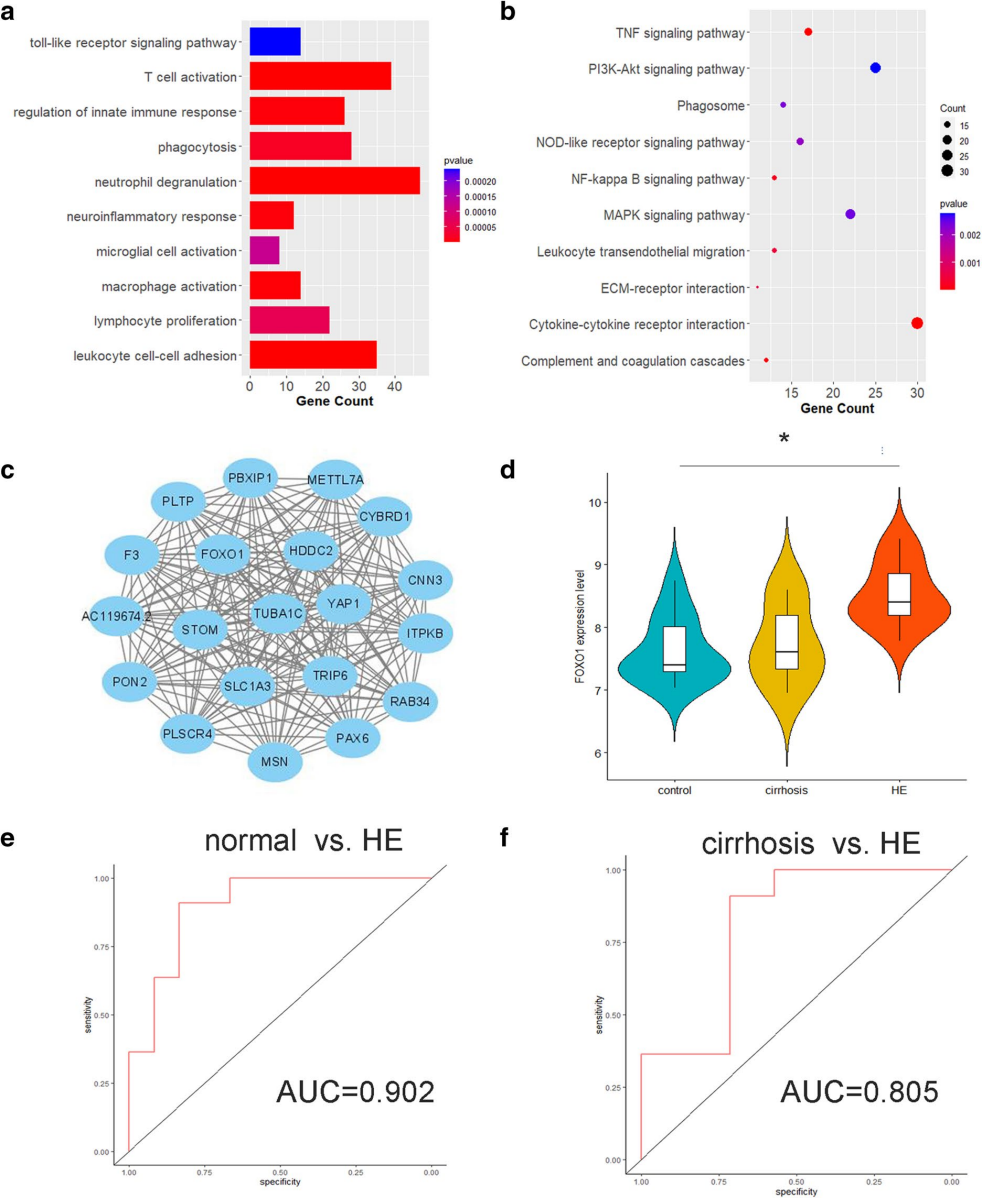
Functional enrichment analysis and hub genes identification of the green module. **a** Bar chart of the top 10 significant terms of BP in GO enrichment analysis of the green module. **b** Bubble diagram of the top 10 enriched pathways in KEGG pathway enrichment analysis of the green module. **c** Network depiction of the green module. The top 20 genes with the highest IC were involved. **d** Violin plots with included boxplots of FOXO1 expression levels among three groups. **e** ROC analysis to evaluate expression level of FOXO1 in predicting cirrhosis. **f** ROC analysis to evaluate expression level of FOXO1 in distinguishing HE from cirrhosis

GO enrichment analysis and KEGG pathway enrichment analysis were utilized to identify the functional enrichment annotations in the green module. The GO enrichment analysis showed that the green module was mainly associated with neuroinflammation-related biological processes (Fig. 4A). For KEGG pathway enrichment analysis, neuroinflammation-, ECM-, and coagulation-related pathways were significantly enriched in the green module (Fig. 4B). To identify the hub gene, the top 20 genes with the highest IC were visualized, which were defined as central genes (Fig. 4C, Additional file 1: Table S2). After constructing the key network, TUBA1C was the gene with the highest degree of connections. However, there was no significant difference among the expression levels of TUBA1C in normal, cirrhosis, and HE groups. Alternatively, FOXO1 was selected as the hub gene of the green module, which had the second highest degree of connections. An elevated expression level of FOXO1 in HE samples was observed compared to healthy control samples (Fig. 4D). Meanwhile, ROC analysis showed that the high expression of FOXO1 could well predict the existence of HE (AUC = 0.902) and distinguish HE from cirrhosis (AUC = 0.805) (Fig. 4E, F).

### Identification of candidate drugs

Based on the central genes in the brown and the green modules, candidate drugs were identified, respectively. For both HE modules, top 10 drug molecules with significant p-values and q-values were shown, which might be potential therapeutic agents against HE. Tamibarotene was the top candidate drug of the brown module with the lowest p-value and q-value (Table 1). Meanwhile, VITAMIN E was identified as the top candidate drug of the green module (Table 2).

**Table 1:** Top 10 significant drugs targeting the genes in brown module

Term	*p*-value	*q*-value	overlap_genes
Tamibarotene CTD 00002527	0.000013	0.003889	[HLA-DMB, ALOX5AP, CYBB, CYBA, LGALS9, AIF1]
leukotriene C4 CTD 00007223	0.000128	0.019335	[SYK, ALOX5AP]
dioxidanide CTD 00006819	0.000620	0.062370	[CYBB, CYBA]
valproic acid HL60 DOWN	0.000921	0.066995	[SYK, PTPN6, LAIR1]
Sodium dichromate CTD 00000827	0.001109	0.066995	[SLC7A7, AIF1, LAIR1]
TACROLIMUS MONOHYDRATE CTD 00007118	0.001519	0.076433	[CYBA, CCR3]
NADP( +) BOSS	0.003302	0.132900	[CYBB, CYBA]
pergolide HL60 UP	0.003521	0.132900	[RBM47, FCGR2A, CYBB]
mebendazole HL60 UP	0.004703	0.157814	[RBM47, FCGR2A, SLC7A7]
Medroxyprogesterone acetate CTD 00006623	0.006509	0.166665	[C1QA, ALOX5AP, AIF1]

**Table 2:** Top 10 significant drugs targeting the genes in green module

Term	*p*-value	*q*-value	overlap_genes
VITAMIN E CTD 00006994	0.000006	0.003297	[ITPKB, TUBA1C, PON2, TRIP6, MSN, STOM, PBXIP1, F3, FOXO1]
anisomycin HL60 DOWN	0.000041	0.011218	[ITPKB, PON2, MSN, CYBRD1, STOM, PBXIP1, METTL7A]
quercetin HL60 DOWN	0.000259	0.028065	[PON2, METTL7A]
wortmannin CTD 00000504	0.000397	0.028065	[YAP1, F3, FOXO1]
rifabutin PC3 UP	0.000438	0.028065	[PLSCR4, STOM, FOXO1]
VALPROIC ACID CTD 00006977	0.000529	0.028065	[YAP1, HDDC2, PON2, SLC1A3, CYBRD1, PAX6, PBXIP1, F3, FOXO1, ITPKB, RAB34, PLSCR4, TRIP6, STOM, METTL7A, PLTP]
Medroxyprogesterone acetate CTD 00006623	0.000533	0.028065	[SLC1A3, STOM, F3, FOXO1]
sodium benzoate BOSS	0.000620	0.028065	[PON2, F3]
2,6-Di-tert-butyl-4-methylphenol CTD 00005548	0.000724	0.028065	[PON2, F3]
MS-275 PC3 UP	0.000783	0.028065	[PLSCR4, STOM, F3, FOXO1]

### Changes in glial molecular markers

**Fig. 5:**
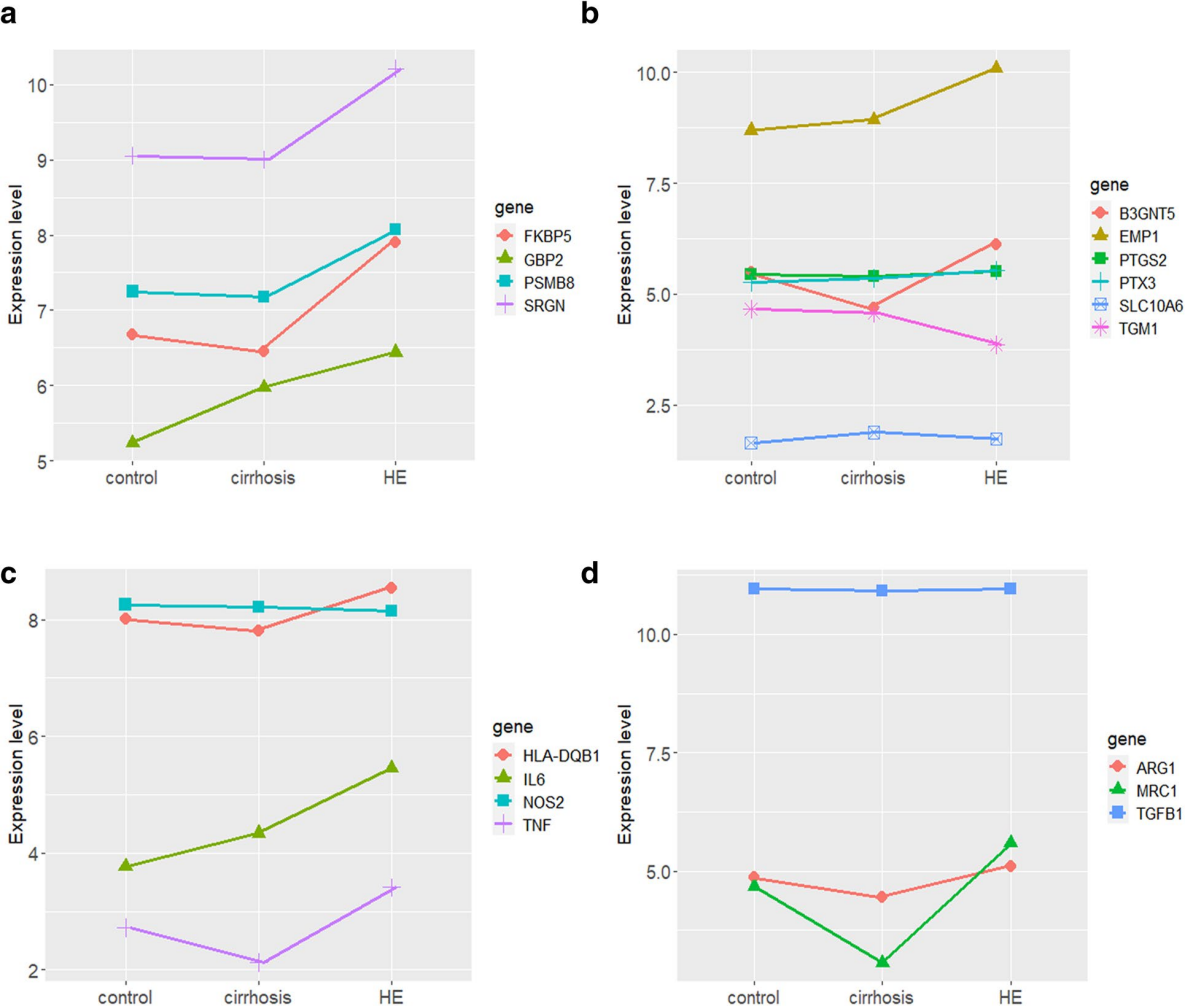
Changes in glial molecular markers among three groups. **a** Line chart of A1 reactive astrocyte-specific markers. **b** Line chart of A2 reactive astrocyte-specific markers. **c** Line chart of M1 microglia-specific markers. **d** Line chart of M2 microglia-specific markers

Overwhelming evidence has suggested the important role of glial cell activation during the pathogenesis of HE [8, 13, 14]. Therefore, after we obtained a thorough picture of the gene expression patterns in HE, further analysis was conducted to investigate the specific expression patterns of astrocyte activation and microglial polarization. Firstly, we adopted specific markers of reactive astrocyte subpopulations, which could represent the A1 and A2 subtypes, respectively [15, 16]. The expression levels of the astrocyte-specific markers among three groups were shown by line charts (Fig. 5A, B). We could observe that the A1 subtype-related markers (FKBP5, GBP2, PSMB8, and SRGN) were remarkably elevated in HE samples compared to healthy control samples and cirrhosis samples (Fig. 5A). On the other hand, the A2 subtype-related markers exhibited mixed patterns of changing (Fig. 5B). Only TGM1 showed a downward trend, while PTGS2, PTX3, and SLC10A6 did not show notable changes. Interestingly, B3GNT5 and EMP1 were upregulated in HE samples, which was probably a compensatory upregulation. Meanwhile, the polarization status of microglia was also investigated. The microglia-specific markers for M1 and M2 subtypes were selected to represent the subpopulations of microglia [16]. According to the line chart, it was obvious that most of the markers in the M1 group (HLA-DQB1, IL-6, and TNF) were upregulated in HE samples. Only NOS2 exhibited a slightly downward trend (Fig. 5C). As for the markers in the M2 group, only MRC1 was elevated in HE samples, especially when compared to cirrhosis samples. ARG1 and TGFB1 did not show notable changes among three groups (Fig. 5D).

### Hub genes verification and GSEA in the rat model of HE

**Fig. 6:**
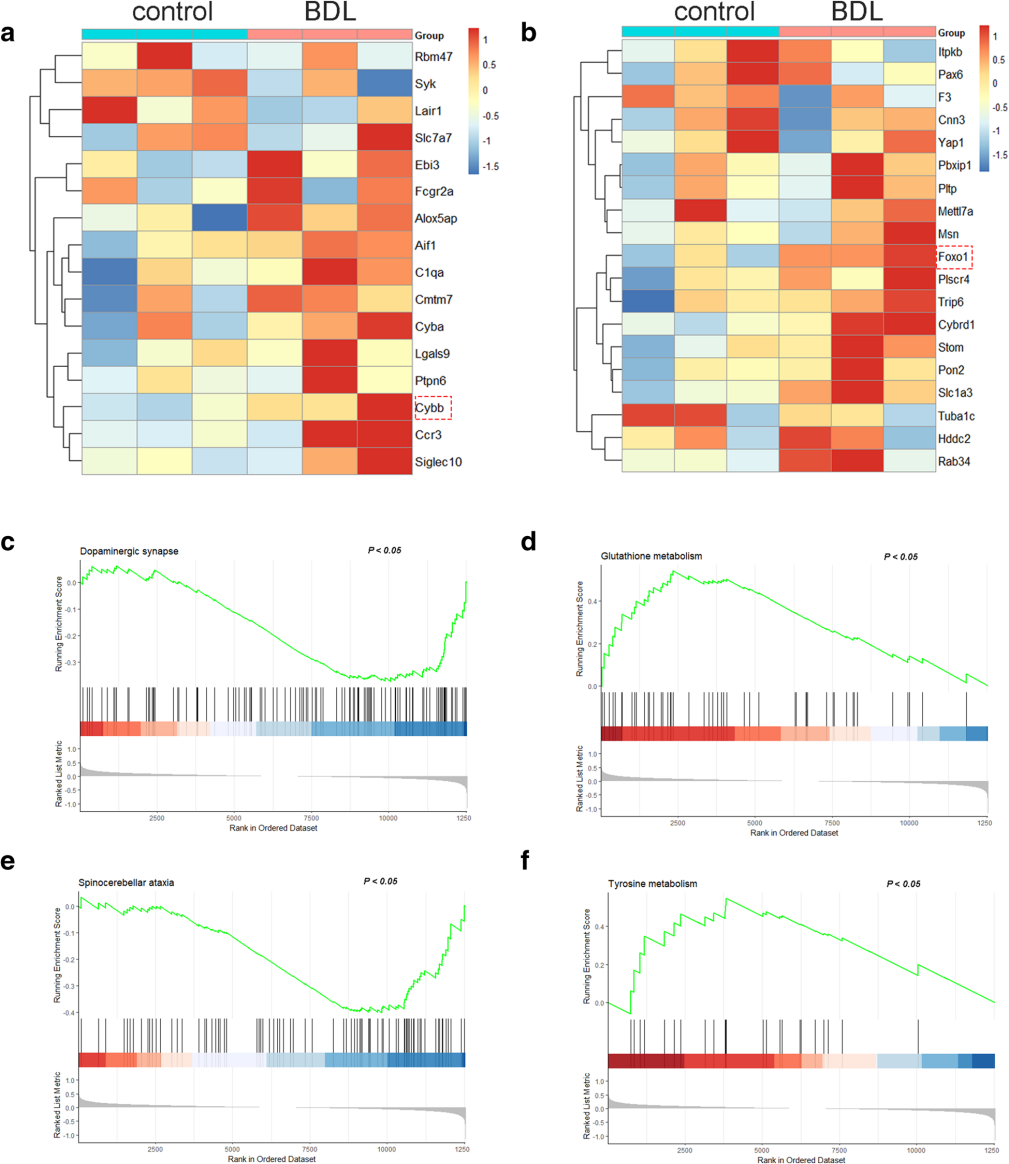
Hub genes verification and GSEA in BDL rats. **a** The heatmap of the central genes in the brown module between control and BDL groups in GSE149227. *n* = 3 (control) and *n* = 3 (BDL). **b** The heatmap of the central genes in the green module between control and BDL groups in GSE149227. *n* = 3 (control) and *n* = 3 (BDL). **c**–**f** Gene sets enriched in the brains from BDL rats by GSEA

To verify the hub genes in the rat model, HE in rats was induced by BDL. Based on transcriptomic data of the rat brains, we compared the expression levels of the central genes in two HE modules. Four central genes in the brown module and one central gene in the green module were not identified in RNA sequencing that might be due to low expression level. According to the heatmaps, both CYBB and FOXO1 were significantly upregulated in the brains of BDL rats (Fig. 6A, B). The upregulation of CYBB and FOXO1 was further validated by RNA sequencing of the brains of BDL rats (Additional file 1: Figure S5A, B). Moreover, GSEA was performed to figure out the potential functions. The gene sets of dopaminergic synapse and spinocerebellar ataxia were enriched and downregulated, while glutathione metabolism and tyrosine metabolism were enriched as upregulated gene sets in BDL rats (Fig. 6C–F).

## Discussion

In the current study, we found that neuroinflammation played a pivotal role in the pathogenesis of HE. CYBB and FOXO1 might be major regulators during the development from cirrhosis to HE. Moreover, microglia and astrocytes were activated to a pro-inflammatory and neurotoxic status, which facilitated the progression of HE. To the best of our knowledge, it was the first time to use WGCNA to analyze brain transcriptomic datasets from human, which emphasized the importance of neuroinflammation in HE. Furthermore, our study provided the first evidence that CYBB and FOXO1 might be potential biomarkers and therapeutic targets of HE in cirrhotic patients.

In the functional enrichment analysis, our results suggested a remarkable overlap of enriched biological processes and signaling pathways between two HE modules. Interestingly, we also observed that the brown module was negatively correlated with cirrhosis, while the green module was negatively correlated with healthy control. Taken together, we could conclude that there were similar underlying mechanisms during the development of cirrhosis and the development from cirrhosis to HE, including neuroinflammation, neuroimmune, ECM, and coagulation cascade.

According to our results, the neuroinflammation in HE was mainly associated with inflammatory cytokine release, macrophage activation, and inflammatory signaling pathways. Overwhelming evidence has suggested the vital role of neuroinflammation during the pathogenesis of HE [17, 18]. Our previous study also highlighted the importance inflammation by functional enrichment analysis based on lncRNAs in the serum transcriptome of HE, which showed similar results as those in the current study [6]. Moreover, Balzano et al. has reported the protective effects of anti-TNF-α treatment against inflammatory cytokine release and microglial activation in the rat brains [19]. We also found that VEGF-C overexpression could alleviate neuroinflammation by enhancing meningeal lymphatic drainage, which eventually improved HE in cirrhotic rats [8]. Therefore, it was obvious that neuroinflammation was a major contributor to the progression of HE. Anti-inflammatory treatments could be effective therapeutic strategies.

In addition to the significant role of neuroinflammation in HE, the importance of neuroimmune, ECM, and coagulation cascade should not be neglected. It was reported that immune paralysis, defined by decreased HLA-DR expression on monocytes, was associated with overt HE (OHE) in liver cirrhosis and ACLF [20, 21]. Moreover, elevated serum levels of ECM-associated proteins were observed in HE, including osteopontin and 7S domain of type IV collagen (4COL7S) [22, 23]. Otherwise, as coagulopathy was a common comorbidity of liver cirrhosis, disturbed coagulation in HE might be attributed to liver insufficiency [24, 25]. Interestingly, the serum level of 4COL7S was also correlated to coagulopathy in ALF, suggesting a complicated regulatory network underlying the pathophysiology of HE [23].

Following the functional enrichment analysis, we performed further analysis in HE modules. In the brown module, CYBB was regarded as the hub gene. CYBB, also known as gp91-phox, is the catalytic subunit of the phagocyte-like NADPH oxidase (NOX2) [26]. Genetic deletion and overexpression of CYBB could modulate the expression of NOX2 [27–29]. In another word, the expression level and catalytic activity of NOX2 largely depended on the expression of CYBB. The upregulation of CYBB we observed in our study suggested an upregulation of NOX2 in the brain of HE patients. Therefore, our results showed that brain NOX2 played an essential role in the pathogenesis of HE. Previous in vitro studies have demonstrated that ammonia could induce the activity of NOX family in astrocytes, which could be reversed by NOX inhibitor apocynin [30, 31]. Moreover, reduction of ammonia by ornithine phenylacetate also led to a decrease of brain NOX1 in BDL rats [32]. However, to the best of our knowledge, the direct relationship between NOX2 and HE has never been reported. NOX2 could mediate neuroinflammation in various CNS disorders, when it most prominently affected astrocytes and microglia via ROS generation [33–36]. Thus, we could speculate that brain NOX2 was an important positive regulator of oxidative stress in HE, which exacerbated neuroinflammation. Interestingly, our GSEA in BDL rats also highlighted an upregulation in glutathione metabolism, which was an essential biological process involved in oxidative stress. Furthermore, various kinds of NOX2 inhibitors have been developed [37, 38]. The protective effects of NOX2 inhibitors against neuroinflammation have been revealed, while the microglial cell was a common target [39–41]. Thus, NOX2 could be a potential therapeutic target of HE, which warranted further translational study.

In the green module, the transcription factor FOXO1 was identified as the hub gene, which was highly expressed in HE samples and BDL rats. As an important transcriptional activator, FOXO1 played a vital role in metabolism modulation, immune response, cell fate decisions, etc. [42]. In CNS disorders, FOXO1 could balance energy metabolism, activate neuroinflammation, induce autophagy and exert anti-oxidative effects [43–45]. However, both upstream and downstream regulatory mechanisms of FOXO1 in HE remain unclear. Previous study highlighted the importance of FOXO1 phosphorylation to modulate the transcriptional activity, when PI3K/Akt and MAPK signaling pathways were significantly enriched in the green module [46, 47]. For the downstream regulation of biological processes, FOXO1 was a transcriptional activator of GSDMD gene in microglia, which enhanced microglia pyroptosis and neuroinflammation [48]. On the other hand, the elevated FOXO1 in HE might be an anti-oxidative signal, when FOXO1 was considered as a sensing element in anti-oxidative signaling [42]. However, contradictory results have been observed for the role of FOXO1 in oxidative stress [42, 49–51]. Therefore, loss-of-function experiments were required to figure out the exact role of FOXO1 in HE. CUT&Tag profiling or chromatin immunoprecipitation sequencing might help to identify the transcriptional targets. Moreover, FOXO1 could directly regulate tyrosine hydroxylase in dopaminergic neurons, when GSEA in BDL rats suggested the alterations of dopaminergic synapse and tyrosine metabolism in HE [52]. Furthermore, potential translational value of FOXO1 should not be neglected. Targeted interventions might affect various related biological processes regulated by FOXO1. Several therapies antagonizing FOXO1 were under research in cancers and metabolic disorders [53]. However, for the clinical application of FOXO1-targeted therapy in HE, there is still a long way to go.

Additionally, tamibarotene and vitamin E were identified as the peak drug candidates of two HE modules, respectively. Overwhelming evidence has suggested that vitamin E was an anti-oxidant, which could alleviate oxidative stress and astrocyte swelling induced by ammonia in vitro [54, 55]. Animal studies also highlighted the anti-oxidative effects of vitamin E to protect against TAA-induced acute HE in the rat model [56, 57]. Furthermore, the combination of vitamin E with other vitamins and zinc could significantly improve HE in cirrhotic patients [58]. Tamibarotene, also known as Am80, was a retinoic acid receptor (RAR) agonist to treat acute promyelocytic leukemia (APL) [59]. Tamibarotene could protect dopaminergic neurons from microglia-mediated inflammatory damages [60]. Previous clinical studies have also reported the anti-inflammatory effects of tamibarotene in CNS disorders including Alzheimer’s disease and intracerebral hemorrhage [61, 62]. However, the effects of tamibarotene on HE remained unknown, which warranted further clinical trials.

Furthermore, it was worth mentioning that the vital role of glial cells in HE has already been uncovered, especially for microglia and astrocytes [8, 14]. Activated microglial cells were capable of inducing neurotoxic reactive astrocytes in neuroinflammation [15]. Thus, we also investigated the changes in glial markers, which showed a remarkable upregulation of M1- and A1-specific markers. On the other hand, the changes in M2- and A2-specific markers exhibited mixed patterns. In conclusion, our results revealed that glial cells were polarized to pro-inflammatory and neurotoxic statuses during the pathogenesis of HE, which further emphasized the importance of glial cells in HE.

The current study does have some limitations. Firstly, due to the lack of clinical characteristics, we could not identify the hub genes related to those more specific clinical parameters including clinical grading and the psychometric hepatic encephalopathy score (PHES). Secondly, gain- and loss-of-function experiments in animal models or cultured cells were also needed to further validate the importance of the hub genes in HE. Thirdly, the gene expression profiles for WGCNA were measured in post-mortem brain samples from human. There were confounding factors that would influence the consistency of sequencing results among the samples.

## Conclusions

In our study, we have identified two clinically significant modules related to HE based on WGCNA, and confirmed the important role of neuroinflammation and neuroimmune in the development of HE. In addition, CYBB and FOXO1 were identified as hub genes and highly expressed in patients with HE. Tamibarotene might be a potential therapeutic agent against HE. Finally, we hope the current study could provide further evidence and ideas for future investigations in targeted therapy of HE.

## Materials and methods

### Dataset download and pre-processing

The HE expression profiles and the corresponding clinical information of the GSE41919 and GSE57193 datasets were systemically extracted from the GEO (http://www.ncbi.nlm.nih.gov/geo/) database. The probe IDs were converted into gene symbols. Expression values of genes corresponding to multiple probes were calculated by averaging the probe values. For the missing data in the gene expression matrices, imputed data were estimated by k-nearest neighbor (KNN) approach with a k = 10 [63]. The ComBat package was utilized to remove the batch effect and merge them into a single dataset for further analysis [64]. The merged dataset containing 31 samples consisted of three clinically defined groups: healthy control (*n* = 12), cirrhosis (*n* = 7), and HE (*n* = 12). According to cluster analysis, one sample (GSM1027458) was removed as an outlier (Additional file 1: Figure S2). Finally, a total of 30 samples were involved in the construction of co-expression network and further analysis, which included 12 healthy control samples, 7 cirrhosis samples, and 11 HE samples.

### Construction of co-expression network

WGCNA R package were used to construct the weight gene co-expression network [12]. Instead of using all 16,416 annotated genes to construct the co-expression network, we selected a quarter of the genes with the greatest variance. A total of 4104 genes were finally involved in the WGCNA. Firstly, after the removal of the outlier, a correlation matrix was constructed according to Pearson’s correlations between all pairwise genes. A proper weighting coefficient β was selected to satisfy the scale-free topology. Then the adjacency matrix was constructed based on the correlation matrix with the weighting coefficient β. The adjacency matrix was subsequently transformed to topological overlap matrix (TOM). The average linkage hierarchical clustering was utilized to identify the genes with similar expression patterns and obtain hierarchical clustering tree based on TOM-based dissimilarity (dissTOM). Then the modules were identified by dynamic tree-cutting method with a minimum size of 30. Furthermore, the module eigengenes (MEs) of each module were calculated, which were defined as the first principal components and representative of the overall expression levels of the corresponding modules. Cluster analysis was performed based on the average distance between modules, which was determined by the Pearson’s correlation analysis between MEs of divided modules. According to the cluster analysis, the modules with high similarity were merged with a merging threshold function at 0.25 [65].

### Identification of clinically significant modules

After merging the modules with high similarity based on the average distance between the MEs of each module, the MEs of the modules were calculated again. The Pearson’s correlation analysis was conducted between each clinical traits and the MEs of each module. *P* < 0.05 was considered as a significant correlation between the module and the clinical trait. Moreover, gene significance (GS) was determined by Pearson’s correlation analysis between the clinical traits and the expression levels of each gene in the modules, respectively [12]. The mean absolute values of GS in each module were calculated to represent the correlation between the module and the clinical trait in another way.

### Identification of hub genes

Hub genes were defined as the genes highly interconnected to other genes of the module. To identify the hub genes, module membership (MM) was calculated to evaluate the correlation between genes and modules. MM was determined by the correlation between the expression levels of genes and the MEs of each module [5]. And the intramodular connectivity (IC) was determined by the sum of the correlation coefficients with other genes in the given molecule. A gene with a higher IC tended to have higher MM values, which suggested a more decisive role in the given module [66]. The top 20 genes with the highest IC were selected as central genes for further analysis. The central genes and the connections among them were subsequently imported into Cytoscape 3.8.0 to visualized the key networks. Finally, the hub gene was defined as the gene of the highest degree within the key networks.

### Identification of candidate drugs

According to the top 20 genes with the highest IC in two HE modules, drug compounds recognition was performed, respectively, to identify the drugs with potential therapeutic effects on HE. Candidate drugs were predicted with the Drug Signatures Database (DSigDB) on the Enrichr platform (https://maayanlab.cloud/Enrichr/) [67, 68]. The final results were generated with p-values indicating the correlation of the gene with the predicted drug. The drugs were deemed as having potential therapeutic effects according to the p-values.

### Functional enrichment analysis of clinically significant modules

Functional enrichment analysis including gene ontology (GO) enrichment analysis and Kyoto encyclopedia of genes and genomes (KEGG) pathway enrichment analysis were performed to investigate the biological functions and related signaling pathways of the genes within the clinically significant modules. Based on clusterProfiler package, central genes with MM > 0.6 were selected for functional enrichment analysis [69]. The cut-off criteria for identification of significantly enriched GO annotations and KEGG pathways were set as *P* < 0.05.

### Transcriptomic analysis of BDL rats

The transcriptomic dataset of the brains from BDL rats (GSE149227) was downloaded from the GEO database [8]. FastQC software was used for quality control checks on raw fastq files. The reference genome of rats (RGSC 6.0/rn6) was acquired from the database at the University of California, Santa Cruz (https://genome.ucsc.edu/cgi-bin/hgTables). HISAT2 was utilized for the construction of genome index and the alignment of reads to the reference genome. The featureCounts command was used to calculate the number of fragments mapped to corresponding genes. Subsequently, based on the processed RNA-sequencing data, DESeq2 package was utilized to compare the expression levels of the central genes in the clinically significant modules between control group and BDL group [70]. The heatmaps were drawn by pheatmap package. Meanwhile, gene set enrichment analysis (GSEA) was also conducted by clusterProfiler package [69]. The gene set database consisted of the chemical and genetic perturbations of curated gene sets, biological process, cellular component, and molecular function in the Molecular Signatures Database. The number of permutations was set as 1000, while other parameters were set as default values. The cut-off criteria for identification of the statistically significant gene sets were set as *P* < 0.05. Figures of GSEA were drawn by enrichplot package.

### Statistical analysis

R language 3.4.0 and GraphPad Prism 8.0.1 (San Diego, CA) were utilized for the statistical analysis. All the continuous variables were presented as the mean ± SEM. For two-group comparisons, unpaired Student’s *t* test was used, while one-way ANOVA followed by post hoc Tukey's test were used for multiple comparisons among three or more groups. The correlations were analyzed by the Pearson’s correlation analysis. Receiver operating characteristic (ROC) analysis was implemented using pROC package [71]. *P* < 0.05 indicated statistically significant differences.

## Supplementary Information

**Additional file 1.** Additional figures.

## Data Availability

All the datasets involved in this study are available in the GEO (http://www.ncbi.nlm.nih.gov/geo/) repository.
