# Supplementary material for: Identification of significant modules and hub genes involved in hepatic encephalopathy using WGCNA
Source: Eur J Med Res. 2022 Nov 24;27:264. doi: 10.1186/s40001-022-00898-3 (PMC9685938; doi:10.1186/s40001-022-00898-3)
Supplement: Supplementary file 1 — Additional file 1. Additional figures. [file 40001_2022_898_MOESM1_ESM.pdf]

Figure S1. Heatmap plot depicting the TOM among all selected genes. Lighter color indicates greater overlap, while darker color indicates lower overlap. The dendrograms are along the heatmap plot.

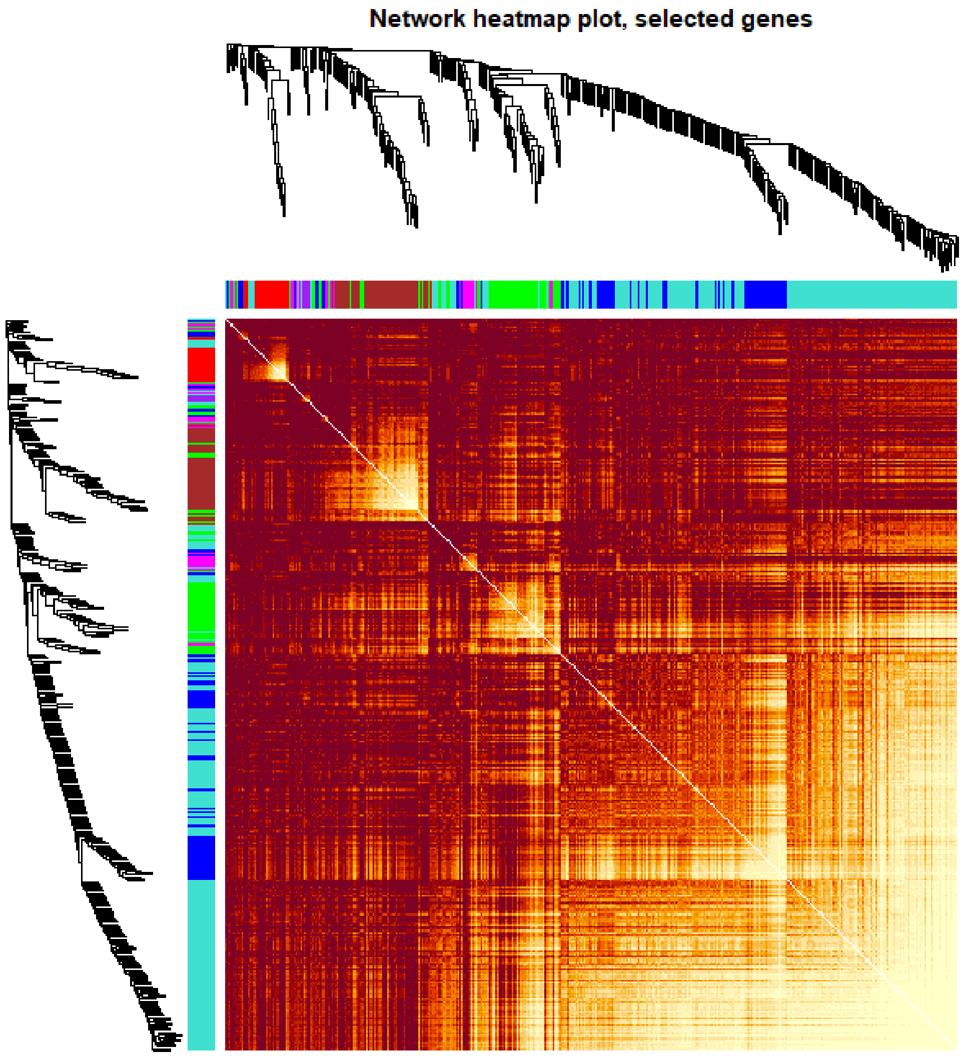

Figure S2. Cluster analysis of all the samples in GSE41919 and GSE57193. GSM1027458 was removed as an outlier.

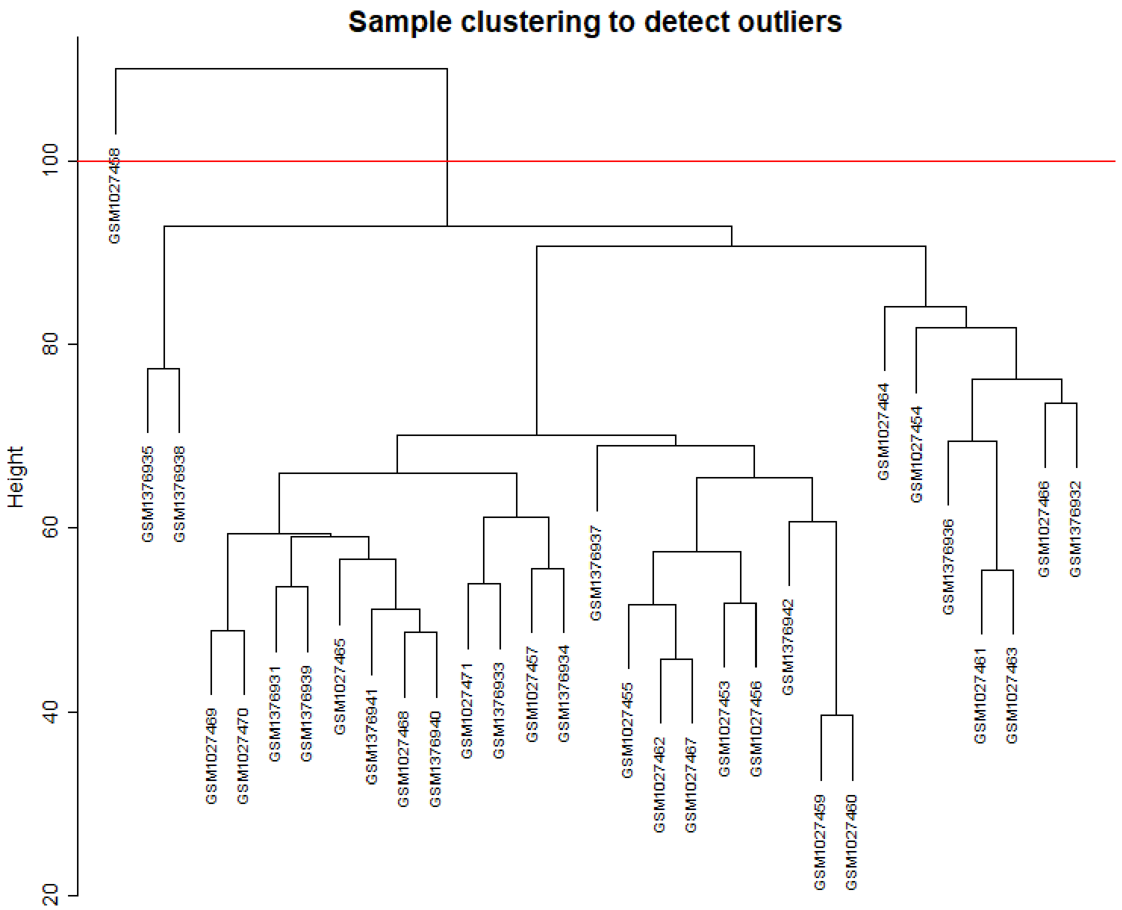

Figure S3. MEs of the corresponding modules. (a-e) The upper plots show the expression levels of all genes in the corresponding modules (y-axis) among all samples (x-axis). The lower plots show the corresponding MEs (y-axis) versus the samples (x-axis).

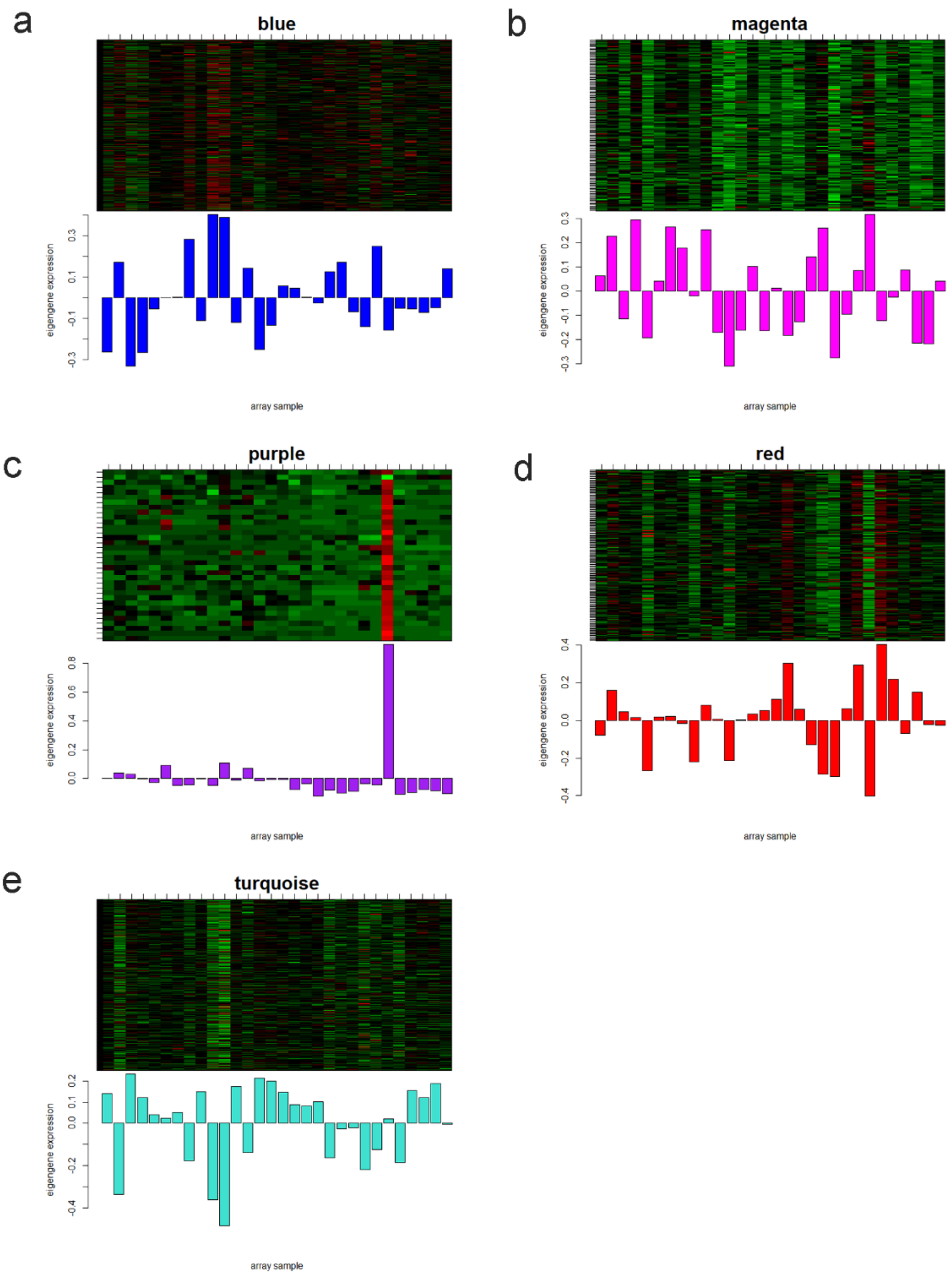

Figure S4. GS for HE versus MM in the corresponding modules. (a-e) Scatterplots of GS for HE versus MM in the corresponding modules.

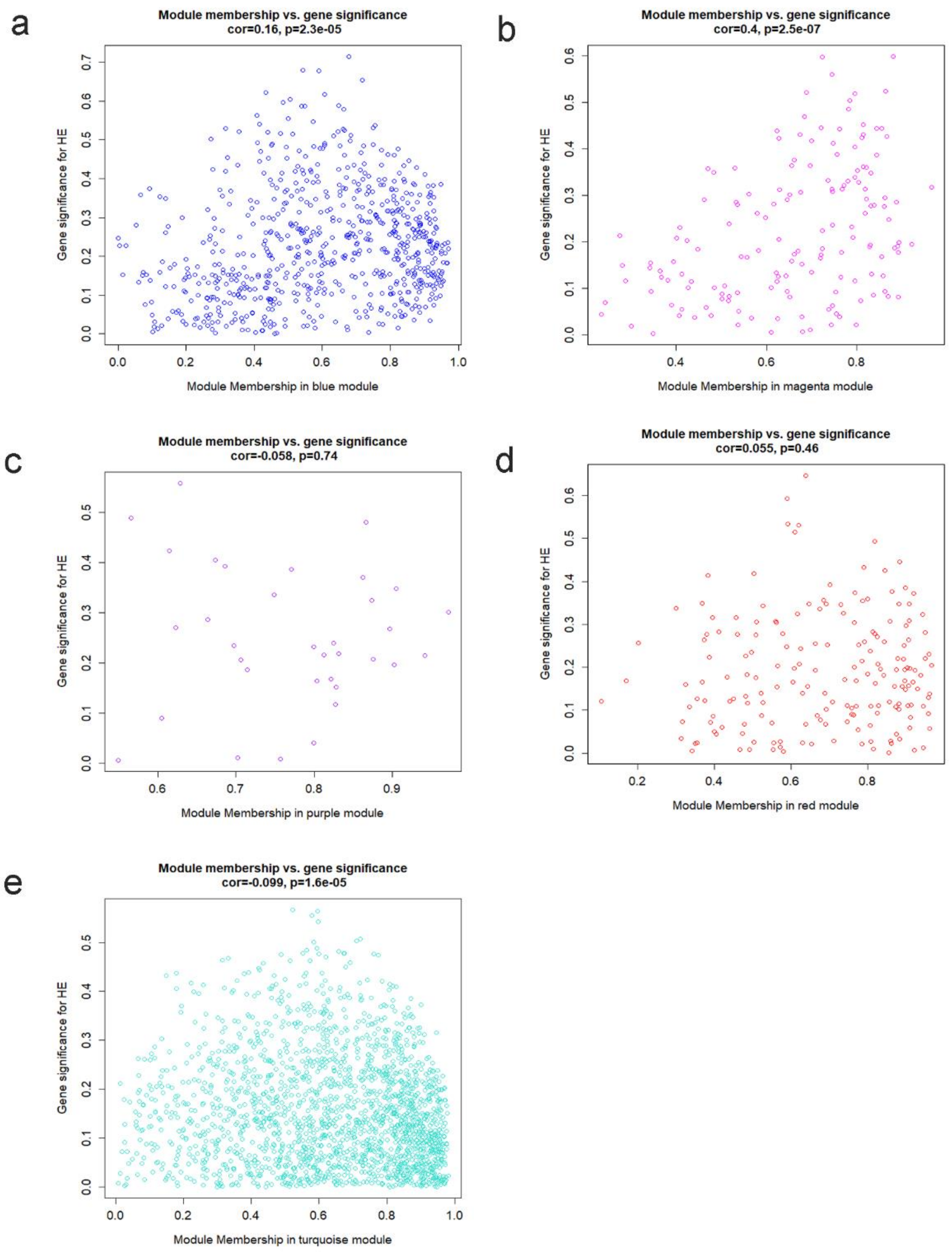

Figure S5. The expression levels of the hub genes in BDL rats by RNA sequencing. (a) Comparison of CYBB levels between Sham control group and BDL group. (b) Comparison of FOXO1 levels between Sham control group and BDL group.

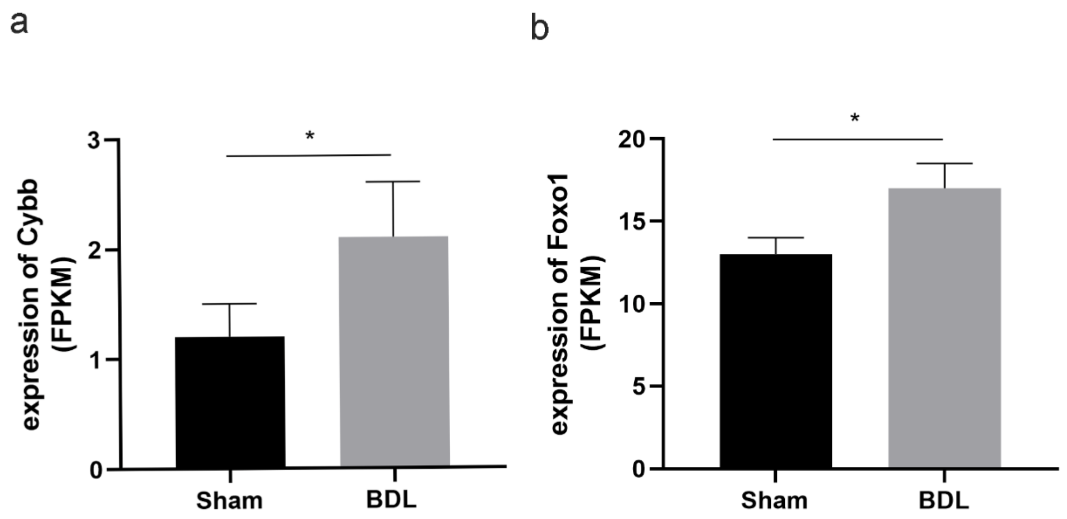

Supplementary materials and methods

Animal model of HE: bile duct ligation

Male Sprague Dawley (SD) rats (6–8 weeks old) were purchased from the Experimental Animal Center of School of Medicine, Shanghai Jiao Tong University (Shanghai, China). The rats were maintained under 22 ◦C, 40–60% humidity, and 12-h light/dark cycle. The rats were fed with standard rat food and had free access to water. All the protocols were approved by the Ethical Committee of Shanghai Ninth People's Hospital, Shanghai Jiao Tong University School of Medicine.

To develop HE in rat, a 4-week bile duct ligation (BDL) model was established as previously described (1, 2). In brief, 8-week-old male Sprague Dawley rats were randomly divided into control group and BDL group, which received sham surgery or common BDL surgery, respectively. The rats received anesthesia with ketamine (75 mg/kg) and xylazine (5 mg/kg) during the surgery, when the common bile duct was ligated with 3-0 silk sutures. The first ligature was placed to the proximal part below the junction of the hepatic ducts, when the second one was placed to the distal portion above the entrance of the pancreatic duct. Then the common bile duct was resected between the ligatures. Vitamin K (50mg/kg) was injected to all rats including sham control group to protected BDL rats from coagulation defects.

RNA sequencing and analysis of rat brains

The RNA-sequencing library preparation was performed as previously described (2). Briefly, total RNA was isolated by the RNeasy Mini Kit (Qiagen), followed by verification of RNA quality and integrity by microvolume spectrophotometry (NanoDrop 2000, Thermo Fisher Scientific) and by on-chip electrophoresis (2100 Bioanalyzer, Agilent Technologies), respectively. The KAPA mRNA HyperPrep Kit (Roche) was used for library preparation. Then the libraries were sequenced via NovaSeq 6000 System (Illumina).

Based on the gene expression profiles from RNA-sequencing, DESeq2 package was utilized to compare the expression levels of the hub genes between sham control group and BDL group (3). The cut-off criteria for identification of the statistically significant gene sets were set as $P < 0.05$.

Reference

1. Hadjihambi A, Harrison IF, Costas-Rodríguez M, Vanhaecke F, Arias N, Gallego-Durán R, et al. Impaired brain glymphatic flow in experimental hepatic encephalopathy. Journal of hepatology. 2019;70(1):40-9.

2. Hsu SJ, Zhang C, Jeong J, Lee SI, McConnell M, Utsumi T, et al. Enhanced Meningeal Lymphatic Drainage Ameliorates Neuroinflammation and Hepatic Encephalopathy in Cirrhotic Rats. Gastroenterology. 2021;160(4):1315-29.e13.

3. Love MI, Huber W, Anders S. Moderated estimation of fold change and dispersion for RNA-seq data with DESeq2. Genome biology. 2014;15(12):550.

Table S1. Key genes of Brown module

Source	Target	Weight
AIF1	AL159163.1	0.360071
AIF1	ALOX5AP	0.392057
AIF1	C1QA	0.383187
AIF1	CCR3	0.368267
AIF1	CMTM7	0.38783
AIF1	CYBA	0.361851
AIF1	CYBB	0.391464
AIF1	EBI3	0.370753
AIF1	FCGR2A	0.363983
AIF1	FYB1	0.398625
AIF1	HCST	0.382397
AIF1	HLA-DMB	0.376906
AIF1	LAIR1	0.381556
AIF1	LGALS9	0.348475
AIF1	PTPN6	0.408599
AIF1	RBM47	0.379522
AIF1	SIGLEC10	0.374492
AIF1	SLC7A7	0.363922
AIF1	SYK	0.391927
AL159163.1	ALOX5AP	0.329864
AL159163.1	C1QA	0.341082
AL159163.1	CCR3	0.334082
AL159163.1	CMTM7	0.327494
AL159163.1	CYBA	0.311027
AL159163.1	CYBB	0.349701
AL159163.1	EBI3	0.336922
AL159163.1	FCGR2A	0.311193
AL159163.1	FYB1	0.342216
AL159163.1	HCST	0.331921
AL159163.1	HLA-DMB	0.343354
AL159163.1	LAIR1	0.332765
AL159163.1	LGALS9	0.299104
AL159163.1	PTPN6	0.34742
AL159163.1	RBM47	0.334772
AL159163.1	SIGLEC10	0.320373
AL159163.1	SLC7A7	0.323418
AL159163.1	SYK	0.350294
ALOX5AP	C1QA	0.362994
ALOX5AP	CCR3	0.368294
ALOX5AP	CMTM7	0.345257
ALOX5AP	CYBA	0.33506

Source	Target	Weight
ALOX5AP	CYBB	0.376005
ALOX5AP	EBI3	0.368737
ALOX5AP	FCGR2A	0.338034
ALOX5AP	FYB1	0.365644
ALOX5AP	HCST	0.367296
ALOX5AP	HLA-DMB	0.358257
ALOX5AP	LAIR1	0.367387
ALOX5AP	LGALS9	0.320313
ALOX5AP	PTPN6	0.357905
ALOX5AP	RBM47	0.372713
ALOX5AP	SIGLEC10	0.349982
ALOX5AP	SLC7A7	0.35759
ALOX5AP	SYK	0.385778
C1QA	CCR3	0.354246
C1QA	CMTM7	0.355498
C1QA	CYBA	0.338952
C1QA	CYBB	0.372124
C1QA	EBI3	0.355937
C1QA	FCGR2A	0.340231
C1QA	FYB1	0.367732
C1QA	HCST	0.350707
C1QA	HLA-DMB	0.361116
C1QA	LAIR1	0.353946
C1QA	LGALS9	0.325685
C1QA	PTPN6	0.377184
C1QA	RBM47	0.354341
C1QA	SIGLEC10	0.348868
C1QA	SLC7A7	0.339959
C1QA	SYK	0.372167
CCR3	CMTM7	0.356958
CCR3	CYBA	0.333436
CCR3	CYBB	0.363007
CCR3	EBI3	0.348798
CCR3	FCGR2A	0.337822
CCR3	FYB1	0.369
CCR3	HCST	0.350264
CCR3	HLA-DMB	0.337659
CCR3	LAIR1	0.357569
CCR3	LGALS9	0.320614
CCR3	PTPN6	0.381052
CCR3	RBM47	0.353356
CCR3	SIGLEC10	0.34972
CCR3	SLC7A7	0.334757

Source	Target	Weight
CCR3	SYK	0.360339
CMTM7	CYBA	0.322939
CMTM7	CYBB	0.370312
CMTM7	EBI3	0.354469
CMTM7	FCGR2A	0.326944
CMTM7	FYB1	0.36233
CMTM7	HCST	0.369506
CMTM7	HLA-DMB	0.366375
CMTM7	LAIR1	0.356046
CMTM7	LGALS9	0.328516
CMTM7	PTPN6	0.349186
CMTM7	RBM47	0.368624
CMTM7	SIGLEC10	0.343583
CMTM7	SLC7A7	0.36189
CMTM7	SYK	0.378225
CYBA	CYBB	0.342458
CYBA	EBI3	0.335995
CYBA	FCGR2A	0.323013
CYBA	FYB1	0.338863
CYBA	HCST	0.350872
CYBA	HLA-DMB	0.327967
CYBA	LAIR1	0.343483
CYBA	LGALS9	0.308962
CYBA	PTPN6	0.330977
CYBA	RBM47	0.349453
CYBA	SIGLEC10	0.334743
CYBA	SLC7A7	0.332876
CYBA	SYK	0.350346
CYBB	EBI3	0.362136
CYBB	FCGR2A	0.34658
CYBB	FYB1	0.384648
CYBB	HCST	0.366307
CYBB	HLA-DMB	0.371436
CYBB	LAIR1	0.366623
CYBB	LGALS9	0.334753
CYBB	PTPN6	0.392265
CYBB	RBM47	0.364528
CYBB	SIGLEC10	0.360112
CYBB	SLC7A7	0.350834
CYBB	SYK	0.384804
EBI3	FCGR2A	0.337951
EBI3	FYB1	0.368113
EBI3	HCST	0.351675

Source	Target	Weight
EBI3	HLA-DMB	0.341485
EBI3	LAIR1	0.360834
EBI3	LGALS9	0.318011
EBI3	PTPN6	0.380362
EBI3	RBM47	0.354193
EBI3	SIGLEC10	0.351991
EBI3	SLC7A7	0.335736
EBI3	SYK	0.368418
FCGR2A	FYB1	0.340428
FCGR2A	HCST	0.353317
FCGR2A	HLA-DMB	0.334358
FCGR2A	LAIR1	0.345243
FCGR2A	LGALS9	0.314209
FCGR2A	PTPN6	0.332539
FCGR2A	RBM47	0.353028
FCGR2A	SIGLEC10	0.333779
FCGR2A	SLC7A7	0.338591
FCGR2A	SYK	0.354725
FYB1	HCST	0.362605
FYB1	HLA-DMB	0.377711
FYB1	LAIR1	0.360807
FYB1	LGALS9	0.329635
FYB1	PTPN6	0.383929
FYB1	RBM47	0.367487
FYB1	SIGLEC10	0.35087
FYB1	SLC7A7	0.357893
FYB1	SYK	0.388261
HCST	HLA-DMB	0.359094
HCST	LAIR1	0.351987
HCST	LGALS9	0.327336
HCST	PTPN6	0.378867
HCST	RBM47	0.357641
HCST	SIGLEC10	0.350196
HCST	SLC7A7	0.348045
HCST	SYK	0.373838
HLA-DMB	LAIR1	0.34932
HLA-DMB	LGALS9	0.33499
HLA-DMB	PTPN6	0.382069
HLA-DMB	RBM47	0.352264
HLA-DMB	SIGLEC10	0.343873
HLA-DMB	SLC7A7	0.345032
HLA-DMB	SYK	0.359022
LAIR1	LGALS9	0.320383

Source	Target	Weight
LAIR1	PTPN6	0.376626
LAIR1	RBM47	0.35841
LAIR1	SIGLEC10	0.349899
LAIR1	SLC7A7	0.342584
LAIR1	SYK	0.374862
LGALS9	PTPN6	0.334975
LGALS9	RBM47	0.328227
LGALS9	SIGLEC10	0.305014
LGALS9	SLC7A7	0.319265
LGALS9	SYK	0.336282
PTPN6	RBM47	0.384502
PTPN6	SIGLEC10	0.358726
PTPN6	SLC7A7	0.372716
PTPN6	SYK	0.399342
RBM47	SIGLEC10	0.352926
RBM47	SLC7A7	0.345523
RBM47	SYK	0.373885
SIGLEC10	SLC7A7	0.33981
SIGLEC10	SYK	0.367375
SLC7A7	SYK	0.360211

Table S2. Key genes of Green module

Source	Target	Weight
AC119674.2	CNN3	0.315652135
AC119674.2	CYBRD1	0.328334649
AC119674.2	F3	0.320200355
AC119674.2	FOXO1	0.30568599
AC119674.2	HDDC2	0.309694145
AC119674.2	ITPKB	0.306808687
AC119674.2	METTL7A	0.339818944
AC119674.2	MSN	0.303190391
AC119674.2	PAX6	0.335329818
AC119674.2	PBXIP1	0.334778998
AC119674.2	PLSCR4	0.313237863
AC119674.2	PLTP	0.313189804
AC119674.2	PON2	0.348115588
AC119674.2	RAB34	0.330798998
AC119674.2	SLC1A3	0.334609674
AC119674.2	STOM	0.327688656
AC119674.2	TRIP6	0.318180177
AC119674.2	TUBA1C	0.281866089
AC119674.2	YAP1	0.328289356
CNN3	CYBRD1	0.304329546
CNN3	F3	0.30805709
CNN3	FOXO1	0.277019266
CNN3	HDDC2	0.309703937
CNN3	ITPKB	0.298450267
CNN3	METTL7A	0.311501866
CNN3	MSN	0.295857811
CNN3	PAX6	0.292860997
CNN3	PBXIP1	0.279396079
CNN3	PLSCR4	0.289222867
CNN3	PLTP	0.289486589
CNN3	PON2	0.302987153
CNN3	RAB34	0.290950787
CNN3	SLC1A3	0.297752623
CNN3	STOM	0.293457012
CNN3	TRIP6	0.288208344
CNN3	TUBA1C	0.270383419
CNN3	YAP1	0.301648998
CYBRD1	F3	0.299150707
CYBRD1	FOXO1	0.290844105
CYBRD1	HDDC2	0.303173293
CYBRD1	ITPKB	0.30365365

Source	Target	Weight
CYBRD1	METTL7A	0.318724188
CYBRD1	MSN	0.30306839
CYBRD1	PAX6	0.315359489
CYBRD1	PBXIP1	0.317936222
CYBRD1	PLSCR4	0.301445011
CYBRD1	PLTP	0.294679455
CYBRD1	PON2	0.339431462
CYBRD1	RAB34	0.31278771
CYBRD1	SLC1A3	0.311428082
CYBRD1	STOM	0.308970854
CYBRD1	TRIP6	0.304651576
CYBRD1	TUBA1C	0.274761298
CYBRD1	YAP1	0.325356007
F3	FOXO1	0.289726503
F3	HDDC2	0.301992478
F3	ITPKB	0.313438752
F3	METTL7A	0.312844537
F3	MSN	0.305743831
F3	PAX6	0.305286555
F3	PBXIP1	0.310650137
F3	PLSCR4	0.296452336
F3	PLTP	0.292773304
F3	PON2	0.33399225
F3	RAB34	0.306413628
F3	SLC1A3	0.301730285
F3	STOM	0.306618103
F3	TRIP6	0.300520546
F3	TUBA1C	0.284554199
F3	YAP1	0.321973963
FOXO1	HDDC2	0.296601111
FOXO1	ITPKB	0.286446638
FOXO1	METTL7A	0.297085409
FOXO1	MSN	0.300465935
FOXO1	PAX6	0.285153541
FOXO1	PBXIP1	0.270167677
FOXO1	PLSCR4	0.27188224
FOXO1	PLTP	0.284587094
FOXO1	PON2	0.309740188
FOXO1	RAB34	0.287049971
FOXO1	SLC1A3	0.282905616
FOXO1	STOM	0.285078647
FOXO1	TRIP6	0.273388477
FOXO1	TUBA1C	0.274345746

Source	Target	Weight
FOXO1	YAP1	0.293668503
HDDC2	ITPKB	0.307901413
HDDC2	METTL7A	0.309413837
HDDC2	MSN	0.311092171
HDDC2	PAX6	0.316312042
HDDC2	PBXIP1	0.314188622
HDDC2	PLSCR4	0.303112754
HDDC2	PLTP	0.3016217
HDDC2	PON2	0.336145121
HDDC2	RAB34	0.321715067
HDDC2	SLC1A3	0.30796456
HDDC2	STOM	0.320535602
HDDC2	TRIP6	0.312595741
HDDC2	TUBA1C	0.296753789
HDDC2	YAP1	0.320110589
ITPKB	METTL7A	0.314604294
ITPKB	MSN	0.280184515
ITPKB	PAX6	0.296053233
ITPKB	PBXIP1	0.308886991
ITPKB	PLSCR4	0.299292831
ITPKB	PLTP	0.286967976
ITPKB	PON2	0.29076467
ITPKB	RAB34	0.296752196
ITPKB	SLC1A3	0.305604597
ITPKB	STOM	0.293208984
ITPKB	TRIP6	0.30096372
ITPKB	TUBA1C	0.283601235
ITPKB	YAP1	0.292806165
METTL7A	MSN	0.304697268
METTL7A	PAX6	0.326725692
METTL7A	PBXIP1	0.336729448
METTL7A	PLSCR4	0.305457879
METTL7A	PLTP	0.300355612
METTL7A	PON2	0.351381954
METTL7A	RAB34	0.321875956
METTL7A	SLC1A3	0.327589769
METTL7A	STOM	0.315881451
METTL7A	TRIP6	0.314470849
METTL7A	TUBA1C	0.276784726
METTL7A	YAP1	0.3366108
MSN	PAX6	0.306148772
MSN	PBXIP1	0.293192294
MSN	PLSCR4	0.307648043

Source	Target	Weight
MSN	PLTP	0.306490099
MSN	PON2	0.290135033
MSN	RAB34	0.311746565
MSN	SLC1A3	0.30360927
MSN	STOM	0.31107206
MSN	TRIP6	0.30342141
MSN	TUBA1C	0.304421107
MSN	YAP1	0.274197173
PAX6	PBXIP1	0.300692461
PAX6	PLSCR4	0.288429324
PAX6	PLTP	0.293909189
PAX6	PON2	0.329270733
PAX6	RAB34	0.304586762
PAX6	SLC1A3	0.309600018
PAX6	STOM	0.301118203
PAX6	TRIP6	0.295063836
PAX6	TUBA1C	0.278148765
PAX6	YAP1	0.311974001
PBXIP1	PLSCR4	0.284142987
PBXIP1	PLTP	0.277878078
PBXIP1	PON2	0.332682764
PBXIP1	RAB34	0.296312093
PBXIP1	SLC1A3	0.315525643
PBXIP1	STOM	0.283681972
PBXIP1	TRIP6	0.288075378
PBXIP1	TUBA1C	0.25222966
PBXIP1	YAP1	0.332302577
PLSCR4	PLTP	0.285500938
PLSCR4	PON2	0.318477332
PLSCR4	RAB34	0.288612418
PLSCR4	SLC1A3	0.291833459
PLSCR4	STOM	0.291203384
PLSCR4	TRIP6	0.281434987
PLSCR4	TUBA1C	0.27811724
PLSCR4	YAP1	0.307634523
PLTP	PON2	0.311849446
PLTP	RAB34	0.298332565
PLTP	SLC1A3	0.285340189
PLTP	STOM	0.30418515
PLTP	TRIP6	0.286540388
PLTP	TUBA1C	0.288120365
PLTP	YAP1	0.290403121
PON2	RAB34	0.328868954

Source	Target	Weight
PON2	SLC1A3	0.338749448
PON2	STOM	0.316131005
PON2	TRIP6	0.317882074
PON2	TUBA1C	0.282284727
PON2	YAP1	0.307588915
RAB34	SLC1A3	0.303853001
RAB34	STOM	0.305190199
RAB34	TRIP6	0.295044105
RAB34	TUBA1C	0.28867416
RAB34	YAP1	0.312524164
SLC1A3	STOM	0.300055134
SLC1A3	TRIP6	0.296247741
SLC1A3	TUBA1C	0.270458237
SLC1A3	YAP1	0.327066251
STOM	TRIP6	0.290912697
STOM	TUBA1C	0.294143974
STOM	YAP1	0.298903377
TRIP6	TUBA1C	0.277535368
TRIP6	YAP1	0.310589874
TUBA1C	YAP1	0.27615963
